# Highly stretchable dynamic hydrogels for soft multilayer electronics

**DOI:** 10.1126/sciadv.adn5142

**Published:** 2024-07-17

**Authors:** Stephen J. K. O’Neill, Zehuan Huang, Xiaoyi Chen, Renata L. Sala, Jade A. McCune, George G. Malliaras, Oren A. Scherman

**Affiliations:** ^1^ Melville Laboratory for Polymer Synthesis, Yusuf Hamied Department of Chemistry, University of Cambridge, Cambridge CB2 1EW, UK.; ^2^ Electrical Engineering Division, Department of Engineering, University of Cambridge, Cambridge CB3 0FA, UK.

## Abstract

Recent progress in the development of synthetic polymer networks has enabled the next generation of hydrogel-based machines and devices. The ability to mimic the mechanical and electrical properties of human tissue gives great potential toward the fields of bioelectronics and soft robotics. However, fabricating hydrogel devices that display high ionic conductivity while maintaining high stretchability and softness remains unmet. Here, we synthesize supramolecular poly(ionic) networks, which display high stretchability (>1500%), compressibility (>90%), and rapid self-recovery (<30 s), while achieving ionic conductivities of up to 0.1 S cm ^−1^. Dynamic cross-links give rise to inter-layer adhesion and a stable interface is formed on account of ultrahigh binding affinities (>10^13^ M^−2^). Superior adherence between layers enabled the fabrication of an intrinsically stretchable hydrogel power source, paving the way for the next generation of multi-layer tissue mimetic devices.

## Teaser

Supramolecular polyionic networks enable highly stretchable, conductive, and self-healing hydrogel devices for bioelectronics.

## INTRODUCTION

Hydrogel machines and devices have begun to emerge at the forefront of bioelectronics and soft robotics, on account of their tissue-like mechanical and electrical properties (*1*–*6*). While conventional electronics use rigid metallic materials with electrons as charge carriers, hydrogel devices use soft water-infiltrated polymer networks with ions as charge carriers, analogous to biological living systems (*7*–*10*). To date, these devices have been demonstrated for use as sensors (*11*, *12*), actuators (*13*, *14*), diodes (*15*–*17*), and power sources (*18*, *19*). A major challenge in designing hydrogel devices for bioelectronic and soft robotics applications is to maintain desirable mechanical properties (high stretchability, compressibility, and tissue-like modulus), while simultaneously achieving high ionic conductivities. High ionic conductivities enable improved signal transduction for bioelectronic recording or stimulation, and increase output currents in hydrogel-based devices (*18*, *20*). Concurrently, superior mechanical properties improve conformability, durability, and biocompatibility when interfacing with dynamic human tissues and when used in soft robotics (*21*, *22*).

Supramolecular polymer networks (SPNs) are particularly promising for fabricating hydrogel devices due to the dynamic nature of their cross-links (*23*, *24*). The reversible association and dissociation of cross-links can dissipate energy within the SPN and endow the materials with superior mechanical properties including high stretchability, compressibility, injectability, and self-healing ability (*25*, *26*). Notably, cucurbit[8]uril (CB[8])–based host-guest interactions have been widely used as supramolecular cross-links owing to the range of accessible binding affinities (10^3^ to 10^13^ M^−2^) (*27*), allowing for highly tunable mechanical and viscoelastic properties in physiological conditions (*28*–*31*). For example, we recently reported highly compressible glass-like networks based on CB[8]-enhanced polar-π interactions (*32*). On account of the substantial deformability and rapid self-recovery, a wearable pressure sensor was fabricated, which showed high sensitivity across a wide range of pressures, paving the way for the use of CB[8]-based SPNs in bioelectronic skins.

However, the intrinsic ionic conductivity of reported SPNs remains low (∼10^−3^ S cm ^−1^) (*21*, *32*–*34*), which has limited their application in hydrogel devices for bioelectronics and soft robotics. The limitation is a result of a lack of mobile ionic species within the network, as most are made using neutral acrylamide-based monomers (*30*, *32*). To increase ion density and retention within the SPN, charged monomers can be used. However, as supramolecular cross-links rely on electrostatic interactions, they are sensitive toward highly ionic mixtures and charge screening effects limit the use of such monomers (*27*, *35*, *36*).

**Fig. 1.**
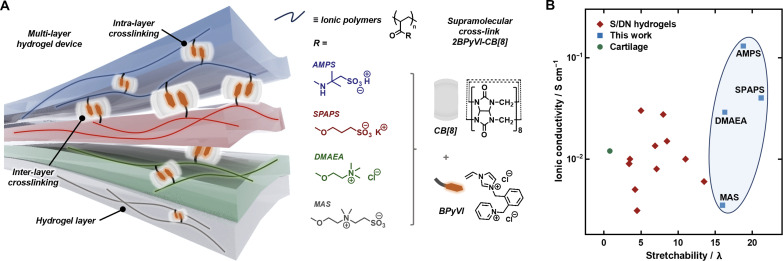
Design of supramolecular polyionic networks. (**A**) Schematic showing intra- and inter-layer supramolecular cross-linking between adjacent poly(ionic) networks on account of the reversible ternary CB[8] complexes. (**B**) Comparison plot of stretchability and ionic conductivity of the SPINs to previous reports.

Here, we report a variety of supramolecular poly(ionic) networks (SPINs) that maintain the superior mechanical properties of SPNs, while achieving ionic conductivities up to as high as 0.1 S cm ^−1^ (Fig. 1A). Furthermore, the dynamic cross-links enable a stable interface to form between distinct hydrogel layers, which has been shown to be critical for the performance and autonomous self-healing of multi-layer stretchable devices (*37*). The formation of a stable interface enables access to a fully stretchable all-hydrogel power source, which can be stretched up to physiologically relevant strains (10 to 50%) while maintaining a stable voltage output.

## RESULTS AND DISCUSSION

We recently reported the synthesis of the molecule BPyVI, which showed high binding affinities with CB[8] (*K*_1_*K*_2_ = 2.3 × 10^13^ M^−2^) and could be incorporated with neutral acrylamide-based polymer backbones (*36*). We hypothesized that such high binding affinity would enable the integration of supramolecular cross-links with ionic polymer materials. To incorporate the supramolecular cross-links within poly(ionic) chains, BPyVI was complexed with CB[8] and copolymerized in situ with ionic monomers via photoinitiated free-radical polymerization (Fig. 1A). The two cationic moieties on the BPyVI guest result in a strong ion-dipole attraction with the CB[8] portal, alleviating charge screening effects from ionic monomers and improving the solubility of the complex (*27*, *35*). The enhanced solubility and ultrahigh binding strength between BPyVI and CB[8] resulted in homogeneous incorporation of the CB[8] cross-links within the network across all concentrations studied (1 to 3 M; figs. S2 and S3 and table S1) (*36*). Conversely, when a singly charged guest monomer BVI was incorporated, an inhomogeneous cloudy network was formed with CB[8] precipitating out of solution (fig. S3).

Free radical copolymerization of the 2BPyVI-CB[8] ternary complex was carried out with a library of different ionic monomers, including anionic [3-sulfopropyl acrylate potassium salt (SPAPS)], zwitterionic {[2-(methacryloyloxy)ethyl]dimethyl-(3-sulfopropyl)ammonium hydroxide (MAS)}, cationic {[2-(methacryloyloxy) ethyl] trimethylammonium chloride (DMAEA)}, and acidic [2-acrylamido-2-methyl-1-propanesulfonic acid (AMPS)] monomers (Fig. 1A). This strategy enables a library of backbones with different charges, yet universal 2BPyVI-CB[8] dynamic cross-links, to achieve a “plug-and-play” system for the fabrication of a variety of hydrogel devices. Pendant BPyVI guest molecules on adjacent polymer backbones bind within the cavity of the CB[8] host molecule in a 2:1 molar ratio, forming an interfacial cross-link between the hydrogel layers.

The dynamic CB[8] host-guest interactions between polymer chains enables high stretchability and compressibility as well as energy dissipation through reversible association and dissociation of the host-guest complexes. Impressively, the strain at break was >15× the original length for each of the SPINs formed, while a broad range of ionic conductivities were observed. Compared to existing reports in the literature, this opens up a combination of mechanical and electrical properties within a single material, previously inaccessible by traditional covalently cross-linked networks (Fig. 1B) (*38*–*48*). Increasing the level of ionic conductivity without sacrificing the stretchability of SPNs improves performance and durability of hydrogel devices, for example, when interfacing between stretchable dynamic human tissues and rigid electrodes, or for soft robotic actuators where materials undergo high local strains (*49*, *50*).

**Fig. 2.**
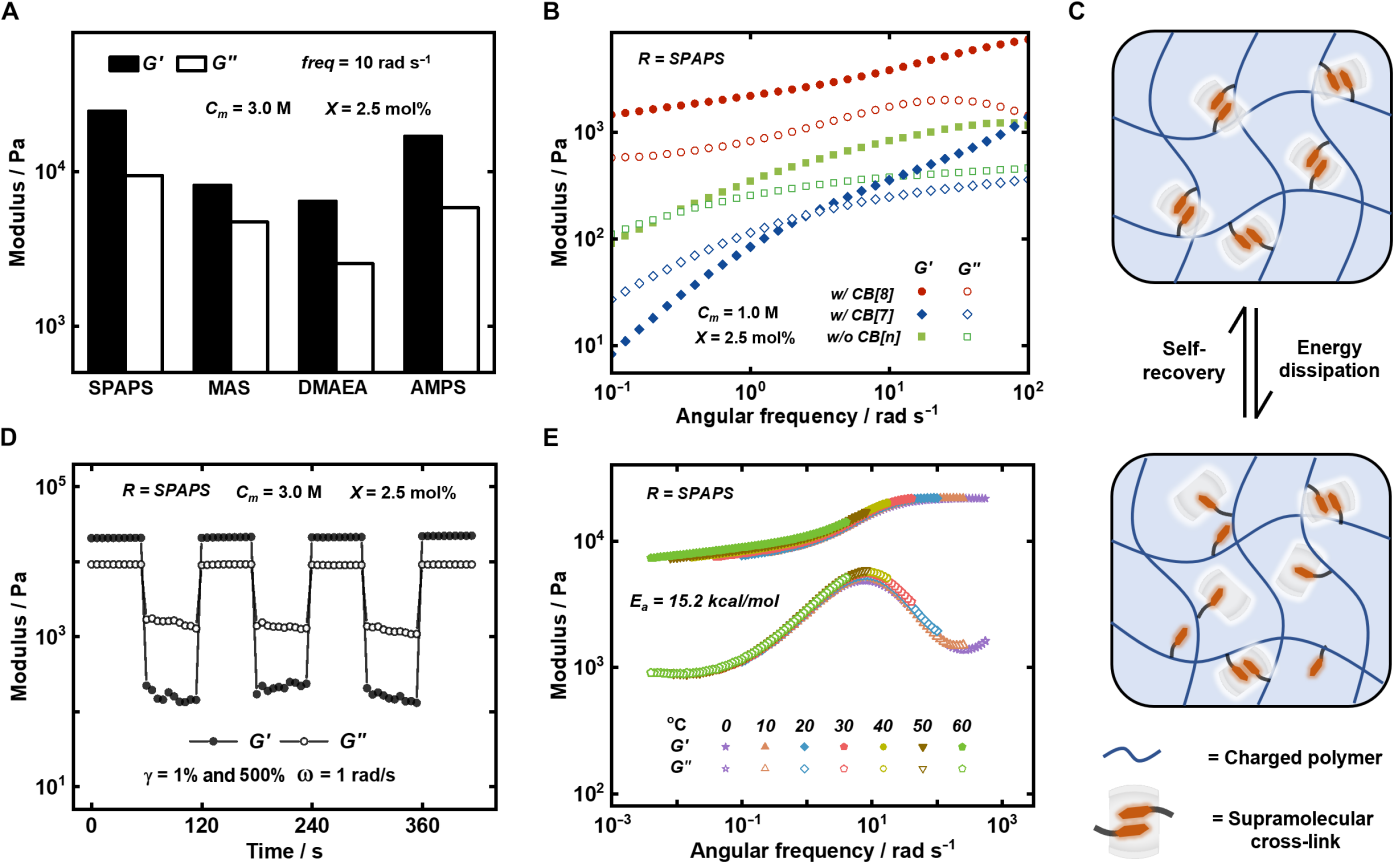
Rheological characterization of the SPINs. (**A**) Histogram plot of *G*′ and *G*″ values for the four SPINs. (**B**) Frequency-sweep measurements at 20°C comparing the supramolecular networks with CB[8], CB[7], and no CB[n]. (**C**) Schematic demonstrating the ternary complexes acting as sacrificial bonds that rupture under deformation and dissipate energy, which can be further reformed, resulting in the self-healing supramolecular network. (**D**) Continuous step-strain measurements at 20°C of the SPAPS networks. (**E**) Master curve of time-temperature superposition for the SPAPS network with dynamic cross-links.

The viscoelastic material properties of the SPINs formed were investigated using oscillatory rheology (Fig. 2A). The zwitterionic (MAS) and cationic (DMAEA) networks show lower moduli compared to anionic and acidic polymers (SPAPS and AMPS). This may be due to ion-dipole interactions between the cationic-containing polymer backbones and the negative dipole at the CB[8] portal, affecting the dynamics of the cross-linking (*27*). To assess the contribution of supramolecular cross-links on the viscoelastic properties of the SPINs, a series of control experiments were performed using the SPAPS monomer to form a model network (Fig. 2B). First, to remove cross-linking between polymer chains, the smaller CB[7] homolog was used as this can only bind one guest within its cavity (fig. S2A). Second, the polymerization was performed in the absence of any CB[n]. The viscoelastic properties of these networks were then studied to elucidate the impact of CB[8] cross-links.

The SPIN formed with CB[8] showed clear gel-like behavior, with considerably higher storage (*G*′) and loss (*G*″) moduli compared to both of the controls, suggesting that CB[8]-mediated cross-linking greatly enhances the strength of the network. Networks formed with CB[7] or without CB[n] demonstrated sol-like behavior at lower frequencies (*G*″ > *G*′). The network formed without CB[n] showed a higher modulus than the CB[7] network. By adding CB[7], weak cross-linking through π − π stacking of the phenyl group in the BPyVI guest is disrupted on account of 1:1 binding (fig. S2) (*30*, *51*, *52*). The disruption of π − π stacking results in a lower *G*′ and *G*″ and a shift toward sol-like behavior with a crossover point at higher frequencies. A range of storage and loss moduli could be accessed by tailoring the SPAPS ionic monomer concentration between 1 and 3 M, while maintaining the cross-linking ratio (*X*) at 2.5 mol% (fig. S5).

The SPINs showed a variety of additional tissue-mimetic properties, including energy dissipation and self-recovery (Fig. 2C). Subjecting the networks to multiple step-strain cycles (1 and 500% strain at 1 rad s ^−1^) led to a reversible sol-gel transition and rapid self-recovery (Fig. 2D). The networks maintained a stable *G*′ and *G*″ over successive strain cycles, somewhat analogous to the self-healing characteristics of human tissue. Energy dissipation within the poly(ionic) networks was investigated through time temperature superposition experiments to obtain the activation energy (*E _a_*) for local chain motion (Fig. 2E). Fitting the temperature data to a horizontal shift parameter (*a _T_*) resulted in an *E _a_* of 15.2 kcal mol ^−1^ (fig. S6), comparable to the unfolding barrier of the I27 domain of the human muscle protein titin (*E _a_* = 17.0 kcal mol ^−1^) (*30*, *53*).

**Fig. 3.**
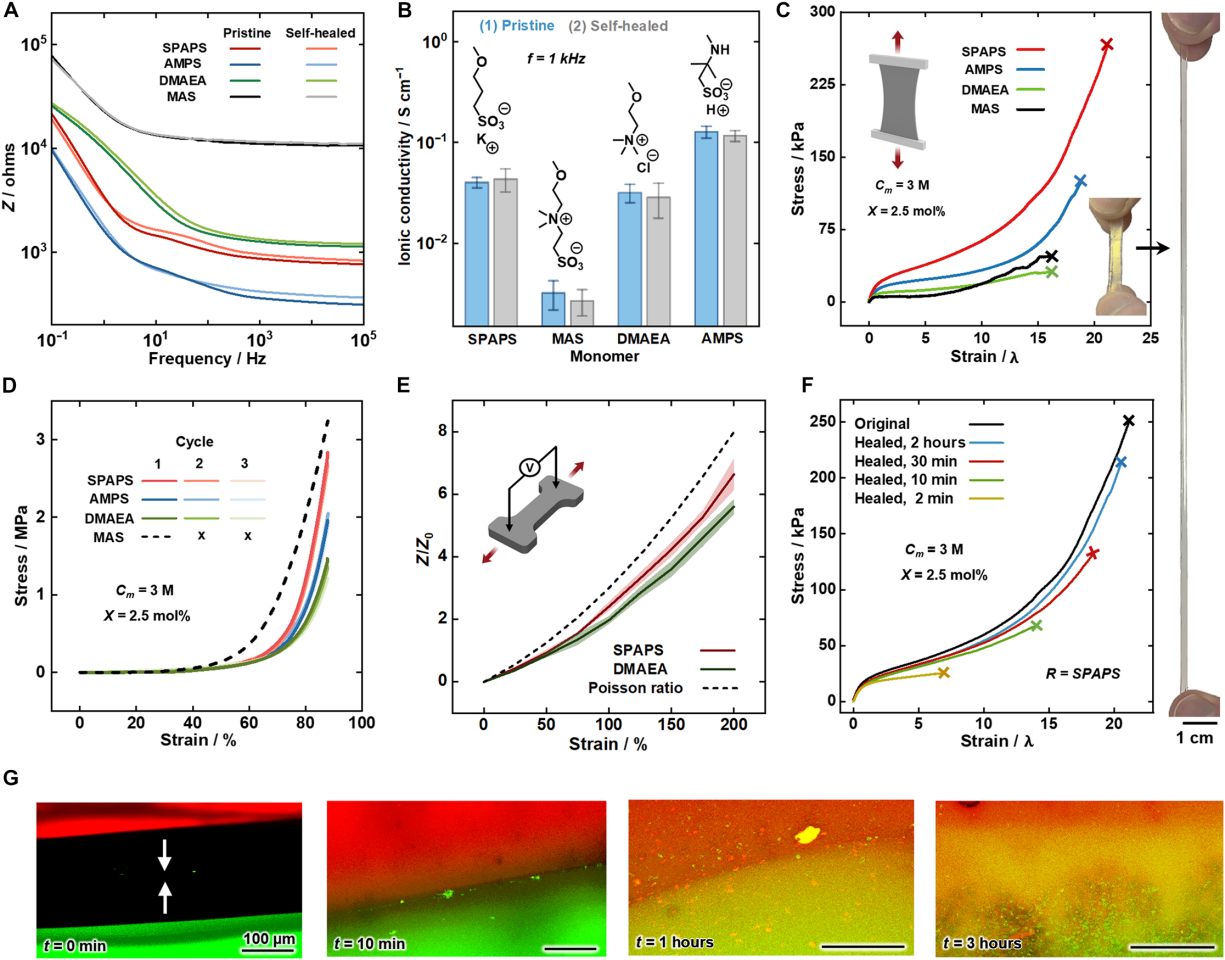
Electrical and mechanical characterization of the SPINs. (**A**) Bode impedance plot comparison of each of the SPINs, showing both pristine and self-healed specimens. (**B**) Ionic conductivity comparison of the four networks, showing pristine (blue) and self-healed (gray) specimens. (**C**) Tensile stress-strain curves of the four SPINs; inset: photographs of a typical SPIN under strain. (**D**) Compressive stress-strain curves for the SPINs over three consecutive cycles. (**E**) Impedance of the SPAPS and DMAEA at 1 kHz during tensile deformation. (**F**) Stress-strain curves of the original and self-healed SPAPS SPIN after different healing times at 25°C. (**G**) Indirect demonstration of self-healing via confocal microscopy of the SPAPS SPIN, one chemically labeled with sulforhodamine B (red), the other with fluorescein (green).

The ionic conductivity of the networks was investigated using electrochemical impedance spectroscopy (EIS), in which the SPINs were placed between two electrodes and an AC voltage was applied at varying frequencies (Fig. 3, A and B). It was found that the zwitterionic polymer (MAS) showed the lowest conductivity, likely due to the lack of a mobile counterion, which is present in the other networks. SPAPS and DMAEA showed a similar ionic conductivity of ∼0.04 S cm ^−1^, while AMPS showed the highest ionic conductivity (∼0.1 S cm ^−1^ at a frequency of 1 kHz), possibly due to the smaller and more mobile hydrogen ion. The effect of self-healing on ionic conductivity was also investigated. Each of the SPINs was cut in half perpendicular to the direction of ionic current and given 30 s to self-heal. Through impedance spectroscopy measurements, no notable difference was observed in ionic conductivity before and after self-healing, suggesting that the self-healed interface does not interfere with ionic mobility across each of the SPINs (Fig. 3A).

We further probed the mechanical properties of the SPINs, through both tensile and compressive testing (Fig. 3, C and D). For both bioelectronic and soft robotic applications, the networks should be mechanically compatible with biological tissues, i.e., compliant and stretchable, as well as robust and tough enough to withstand everyday movement (*3*, *54*). A similar trend in modulus was observed for tensile testing as rheological measurements, with the poly(cationic) chains having a lower modulus, again possibly due to ion-dipole interactions between CB[8] portal and poly(cationic) backbones. All of the networks showed a high degree of extensibility before fracture (<15× initial length), with the cationic SPAPS network displaying more than 20× extensibility of the initial length. Furthermore, to assess the suitability of the networks in a stretchable device, the impedance of the anionic and cationic gels were also tested under tensile deformations up to 200% strain (Fig. 3E). The networks showed a typical impedance response for an ionic conductor, remaining below that of the theoretical Poisson ratio (*1*).

The networks also showed impressive compressibility and subsequent rapid self-recovery (Fig. 3D). The anionic, cationic, and acidic polymer networks showed no fracture at 90% compression and fully recovered within 30 s over three cycles of compression. However, in the case of the zwitterionic polymer network (MAS), water was expelled out of the hydrogel during the compressive test. This may be due to the intra- and interchain ionic interactions from opposing charges on the zwitterionic backbone, driving the polymers toward a coiled structure (*55*), and pushing the water out from the network.

On account of the dynamic and reversible cross-links, the SPINs also showed exemplary self-healing properties tested by cutting a dumbbell-shaped hydrogel sample in half, re-attaching each side, and performing a typical tensile test (Fig. 3F). It was found that within 2 min, the stretchability of the healed hydrogel was already markedly higher than that of human tissue (>500%). Following 2 hours, the hydrogel recovered more than 90% of stretchability compared to the pristine state. While the materials show efficient self-healing ability and high stretchability, there is an intrinsic trade-off with elasticity, which can be observed from extension and retraction tensile curves. A hysteresis loop can be observed, especially at low rates of extension (figs. S9 and S10). The self-healing properties of the hydrogel were also indirectly probed by observing the diffusion of dyes across the healing interface (Fig. 3G). Separate SPAPS gels loaded with either sulforhodamine B (red) or fluorescein (green) were placed in contact, and the diffusion of the dyes at the interface was examined over time using confocal microscopy. It was observed that a yellow color formed at the juncture owing to the diffusion of dyes across the interface, indicating effective self-healing within the gel sample.

**Fig. 4.**
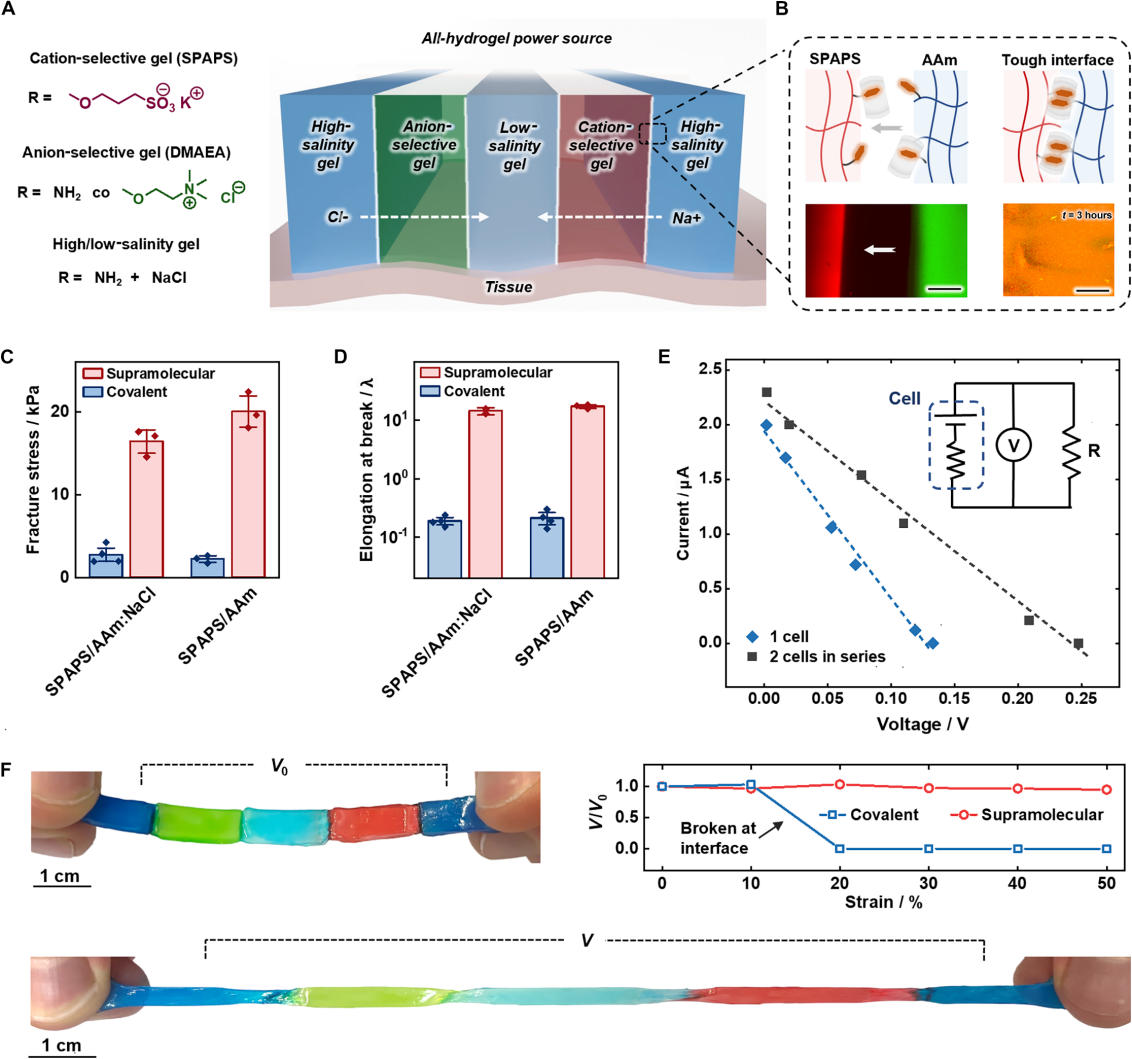
Demonstration of the SPINs in a multi-layer hydrogel power source. (**A**) Design of the soft power source, including a high-salinity hydrogel, a cation-selective gel, a low-salinity gel, and an anion-selective gel. (**B**) Fluorescence microscopic images of the interface between the SPAPS and AAm supramolecular polymer networks, chemically labeled with sulforhodamine B (red) and fluorescein (green). (**C**) Adhesion testing of the SPINs compared to covalently cross-linked networks, showing a bar plot of the fracture stress. (**D**) Adhesion testing of the SPINs compared to covalently cross-linked networks, showing a bar plot of the elongation at break. (**E**) Plot of current and voltage in response to various external loads for one gel cell (black) and two gel cells (blue) in series. (**F**) Photographs and voltage measurements of the hydrogel power source under applied strain.

To demonstrate the applicability of the SPINs in hydrogel devices, we developed a soft power source, which utilizes the poly(anionic) and poly(cationic) networks as cationic and anionic selective membranes. Inspired by the electric eel, it has been demonstrated that by interfacing a high-salinity hydrogel, a cation-selective gel, a low-salinity gel, and an anion-selective gel in sequence, a voltage can be formed via reverse electrodialysis (*18*, *56*–*58*). The salt concentration gradient between the high- and low-salinity gels allows for Cl^−^ ions to pass through the anion-selective gel and Na^+^ ions to pass through the cation-selective gel, resulting in an imbalance of charges across the cell (Fig. 4A). To date, such hydrogel power sources have only used covalent networks and have not been demonstrated under strain on account of poor interfacial stability between layers (*18*, *19*, *59*). We hypothesized that dynamic networks would demonstrate superior interfacial stability through the formation of inter-layer cross-links, achieving a fully stretchable, conformable all-hydrogel power source.

To form the high- and low-salinity gels, acrylamide monomer (AAm, 2 M) was copolymerized with 2.5 mol% 2BPyVI-CB[8] monomer complex in the presence of high (2.5 M) and low (0.015 M) concentrations of NaCl (fig. S12). To ensure even strain application across the hydrogel device with stress, additional acrylamide (1 M) was copolymerized with the DMAEA ionic monomer (2 M) to increase the strength of the anionic-selective hydrogel (fig. S13).

The interfaces formed between different polyionic networks was probed using dye-diffusion experiments as well as adhesion testing (Fig. 4, B to D). Similar to the self-healing test, fluorescein and sulforhodamine B dyes were loaded into polyacrylamide (AAm) and SPAPS gels, respectively, and the diffusion was monitored via confocal microscopy. Faster diffusion was observed compared to just the SPAPS gel alone and, within 3 hours, the dyes were completely mixed at the observation window boundary (Fig. 4B). The faster diffusion of dyes is likely due to ions diffusing from the SPAPS to AAm gel.

To probe the interfacial toughness that supramolecular cross-links can impart between adjacent hydrogel layers, adhesion testing was conducted and compared to covalently cross-linked networks. Testing was performed between the cation-selective hydrogel (SPAPS), and high- and low-salt AAm hydrogels (Fig. 4, C and D). Specimens were cut into rectangular pieces and then brought into contact with the alternative hydrogel, and the force required to detach the two hydrogels at the interface was then measured. It was found that the fracture stress required for the supramolecular gels was more than five times higher than that of their covalent counterpart, suggesting a stronger interface in the supramolecular gel on account of the interfacial association of cross-links between hydrogel layers. The supramolecular cross-links enabled the layered specimens to be elongated by more than 10 times their initial length before fracturing at the interface (Fig. 4D). The adhesion of SPAPS to the low-salinity gel was higher than to the high-salinity gel. This is likely due to the effect of salts on the binding dynamics of the 2BPyVI-CB[8] complex (fig. S11). Through ^1^H nuclear magnetic resonance (NMR) spectroscopy, it was found that at higher salt concentrations, the peaks associated with the guest begin to broaden and shift downfield, suggesting a change in complexation dynamics, which can be attributed to charge screening effects or competitive binding with ionic species.

Interfacing each of the component hydrogels in series, a voltage of ∼115 to 125 mV open circuit was measured, in line with values observed for previously reported covalent eel-inspired hydrogel power sources (*18*). We constructed voltage-power curves by connecting a series of known-load resistances to the power source while monitoring the voltage across the load (Fig. 4E). Currents of up to 2 μA were achieved when low resistances were connected in parallel. In addition, by connecting two cells in series, it was observed that the open circuit voltage scaled linearly with the number of cells, increasing to ∼248 mV. The voltage of the power source was also tested up to physiologically relevant strains (e.g., 50%), which can be experienced by bioelectronic devices under everyday use (Fig. 4F) (*60*). In the case of the SPINS, the voltage remained stable (within 10% of initial value), and the interface was preserved between the hydrogel layers. In contrast, the covalent gel showed an interfacial fracture at only 10% strain, leading to a voltage drop down to zero.

In summary, we have successfully developed a variety of SPINs based on anionic, cationic, zwitterionic, and acidic polymer backbones. By incorporating supramolecular ternary complexes with ultrahigh binding strengths (>10^13^ M^−2^), the reversible association and dissociation kinetics endowed the networks with highly tunable viscoelastic properties, as well as tissue-like self-recovery and energy dissipation. Each of the networks also showed high stretchability (>1500%), while simultaneously achieving high ionic conductivities of up to 0.1 S cm^−1^. Furthermore, the dynamic nature of the cross-links enabled a stable interface to form between different ionic polymer networks. As a result, we developed a fully stretchable, multi-layer all-hydrogel power source that could maintain a stable voltage up to 50% strain. We anticipate that such a combination of electrical and mechanical properties will enable the next generation of multi-layer hydrogel devices for integrated bioelectronic platforms including actuators, organic electrochemical transistors, or electrochromic displays in bioelectronics and soft robotics.

## MATERIALS AND METHODS

### Materials

Unless otherwise stated, all chemicals in this research were purchased from Sigma-Aldrich and used directly without further purification: 1-vinylimidazole (99%), α,α′-dichloro-o-xylene (98%), pyridine (99.8%), acetonitrile (HPLC, 99.9%), diethyl ether (ACS reagent, 99%), ethanol (absolute, 99.8%, HPLC), deuterium oxide (D _2_O, D 99.8%), AMPS (99%), SPAPS (99%), MAS (95%), DMAEA solution (75% in H _2_O, purified through an alumina plug to remove monomethyl ether hydroquinone inhibitor), acrylamide (for molecular biology, 99%, HPLC), sodium chloride (ACS reagent, 99.0%), 2-hydroxy-4-(2-hydroxyethoxy)-2-methylpropiophenone (I-2959, photoinitiator, 98%), and *N,N*′-methylenebis (acrylamide) (99%). CB[8] was prepared according to previously reported procedures (*61*, *62*). Milli-Q water was simply obtained from a Milli-Q Integral Water Purification System (18.2 megohms cm). Unless otherwise noted, all the sample solutions were prepared in D _2_O or Milli-Q H _2_O under heating and ultrasonication.

### Isothermal titration calorimetry

Isothermal titration calorimetry (ITC) experiments were conducted on a Malvern MicroCal Auto-ITC200 apparatus at 298.15 K in Milli-Q H _2_O. In a typical titration, the host molecule (CB[8]) was loaded in the sample cell at a concentration of 0.05 mM, and the guest monomer (BPyVI) was loaded in the syringe at a 20-fold higher concentration of 1.0 mM. One titration experiment consisted of one injection of 0.6 ml and 32 consecutive injections of 1.2 ml with 90-s intervals between injections. The first data point was removed before data analysis as it may be contaminated. The resultant ITC curves were fitted by the sequential binding model, using Malvern MicroCal Analysis Centre software to gain thermodynamic information. All the titrations were repeated three times to provide the mean values of thermodynamic and kinetic parameters with their corresponding error bars (SD, *n* = 3).

### Synthesis of guest monomer BPyVI

α,α′-Dichloro-*o*-xylene (6.66 g, 38 mmol, 1.0 eq.) and pyridine (3.13 ml, 3.07 g, 38.8 mmol, 1.02 eq.) were added to a mixture of ether (20 ml) and chloroform (5 ml) and refluxed for 48 hours. The resulting mixture was transferred to two centrifuge tubes and ether was added (30 ml each), resulting in 1-(2-((1-vinyl-1H-imidazol-3-ium-3-yl)methyl)benzyl)pyridin-1-ium chloride precipitation as a white solid. The white solid was collected as the precipitate with centrifuge at 10,000 rpm for 10 min, washed with ether (re-suspended in 40 ml of ether and centrifuged again to collect the precipitate), and dried in vacuo for use in the next step. 1-(2-((1-Vinyl-1H-imidazol-3-ium-3-yl)methyl)benzyl)pyridin-1-ium (2.92 g, 11.5 mmol, 1.0 eq., 30% yield) and *N*-vinylimidazole (2.24 g, 23 mmol, 2.0 eq.) were added to acetonitrile (30 ml) and refluxed overnight. The resulting mixture was transferred to two centrifuge tubes and precipitated with ether (30 ml each), resulting in 1-(2-((1-vinyl-1H-imidazol-3-ium-3-yl)methyl)benzyl)pyridin-1-ium dichloride (BPyVI) precipitation as a white solid. The white solid was collected as the precipitate with centrifuge at 10,000 rpm for 10 min, washed with ether (re-suspended in 40 ml of ether and centrifuged again to collect the precipitate), and dried in vacuo (2.58 g, 7.4 mmol, 64% yield).

^1^H NMR (400 MHz, D _2_O) δ = 8.67 (d, *J* = 6.2 Hz, 3H), 8.48 (t, *J* = 7.9 Hz, 1H), 7.95 (t, *J* = 7.0 Hz, 2H), 7.67 (s, 1H), 7.44 to 7.28 (m, 3H), 7.54 (q, *J* = 6.3 Hz, 2H), 6.94 (dd, *J* = 15.6, 8.7 Hz, 1H, H _2_O), 5.89 (s, 2H), 5.70 to 5.62 (d, 1H), 5.49 (s, 2H), 5.38 to 5.31 (d, 1H).

^13^C NMR (125 MHz, D _2_O) δ = 144.32, 131.76, 131.64, 131.52, 131.39, 131.09, 130.75, 128.58, 127.98, 122.97, 110.26, 120.03, 120.02, 61.29, 50.43. ESI-MS for {BPyVI·Cl} ^+^: found mass/charge ratio (*m*/*z*) = 312.1262, calculated *m*/*z* = 312.1263.

### Synthesis of SPINs

Certain amounts of ionic monomer, noncovalent cross-linker (2BPyVI-CB[8], 2.5 mol% compared to the ionic monomer concentration), and photoinitiator (I-2959, 0.03 mol% compared to the ionic monomer concentration) were predetermined and dissolved in Milli-Q water under ultrasonication and heating for 30 min. The obtained precursor solution was then sealed and purged with nitrogen for at least 30 min to remove oxygen in the solution phase that may eliminate radicals during polymerization. The precursor solution was carefully injected into a laboratory-made glass mold with a nonstick flourinated polymer lining until the whole mold was filled without any bubbles or spare space inside. The glass mold filled with the precursor solution was exposed to ultraviolet irradiation at 350 nm with 4.8 mW/cm ^2^ for 6 hours to undergo in situ photo-polymerization in one pot. After in situ polymerization, the SPNs were removed from the glass mold and further cut into the test specimens with different sizes and shapes using a dumbbell/cylinder-shaped cutter or a razor blade.

### Rheology

Rheological characterization was carried out by a Discovery Hybrid Rheometer (DHR)-2 (TA Instruments) with a Peltier Plate for temperature control. All the measurements were conducted using a 20-mm parallel stainless steel plate geometry, and the necessary calibration for geometry was carried out before testing. All the samples for rheological characterization were measured just after the E-SPN fabrication. Oscillatory frequency-sweep measurements were conducted at 1% strain in the frequency range from 0.1 to 100 rad s ^−1^. The data were collected at 293.15 K and analyzed by TRIOS software (TA Instruments).

### Mechanical testing

Tensile tests were performed, just after the network synthesis, on a Tinius Olsen Model HK 25-kN Benchtop Tester machine equipped with a 25-N load cell at room temperature. A typical tensile test was performed by stretching a dumbbell-shaped specimen (following ISO4661-1 standard) at 10 mm min ^−1^ (unless otherwise specified) until its breakage, to obtain stress-strain curves. Young’s modulus and toughness are calculated from the slope and the area under the curve, respectively. Extension and retraction cyclic tensile tests were performed using an Instron 3400 series universal testing system (34TM-10).

Compressive tests were conducted on a Tinius Olsen Model HK 25-kN Benchtop Tester machine equipped with a 1-kN load cell at room temperature. In a typical compressive test, a cylinder specimen (∼20 mm diameter × ∼4 mm height) was placed between two stainless steel plates and compressed at a specific deformation rate (10 mm min ^−1^) to obtain stress-strain curves. Compression-retraction cycles were obtained by compressing cylinder specimens until the set strain of 90% and returning to the initial point.

Adhesion testing was performed by cutting rectangular specimens of the hydrogels (5 mm width × 10 mm length × 2 mm height) and contacting two different specimens together at the end points. After 5 min, a typical tensile test was performed, in which the specimen was stretched until the newly joined interface was broken. The fracture stress was taken as the maximum force applied just before the point at which the interface between the hydrogels was ruptured.

### Electrical characterization

To measure impedance, potentiostatic EIS was recorded using a Gamry reference 3000 potentiostat. The hydrogels were cut into rectangular specimens, and AC impedance measurements were obtained between 100 kHz and 500 MHz with an applied amplitude of 20 mV _rms_ relative to the open circuit potential. To measure the electrical properties during deformation, an LCR meter (Compact LCR Meter ST2830) was used to measure the impedance across the stretched E-SPN sample at a fixed frequency of 1 kHz, while the E-SPN was stretched up to 200% strain.

### Confocal microscopy

SPINs loaded with fluorescent dyes and a thickness of 1 mm were imaged using a Leica Stellaris 5 confocal microscope (Leica Microsystems) with a 10× objective and 0.4 numerical aperture. Stacks of 30 images with a resolution of 1024 × 1024 pixels were collected at an excitation wavelength of 559 and 498 nm for hydrogels loaded with sulforhodamine B or fluorescein, respectively. The three-dimensional dataset was processed by Fiji/ImageJ, and maximum-intensity Z-projection images of the hydrogels at different time points during the self-healing process were compared.

### Stretchable power source fabrication and characterization

The hydrogel power source was made by contacting a high-salinity hydrogel, a cation-selective gel, a low-salinity gel, and an anion-selective gel in sequence in series (Fig. 4A). The high- and low-salinity hydrogels were prepared in the same way as the SPINs, with the exception that neutral acrylamide monomer (2 M) was used and certain amounts of NaCl were added. For the high- and low-salinity gels, 2.5 and 0.015 M NaCl were added to the prepolymerization precursor solutions, respectively. The anion-selective gel was prepared using DMAEA (2 M), AAm (1 M), 2BPyVI-CB[8] (0.05 M), and Igacure-2959 photoinitiator (1.2 mM). The cation-selective gel was prepared using SPAPS (2 M), 2BPyVI-CB[8] (0.05 M), and Igacure-2959 photoinitiator (1.2 mM). Gold electrodes were contacted with the first and final high-salinity gel compartments of each series of gels. Voltages were recorded using a FLUKE 115 True RMS multimeter with a high input impedance (>10 M ohms). Current-voltage curves were constructed by connecting a series of known-load resistances to the batteries while monitoring the voltage across the load.
