# Supplementary material for: Highly stretchable dynamic hydrogels for soft multilayer electronics
Source: Sci Adv. 2024 Jul 17;10(29):eadn5142. doi: 10.1126/sciadv.adn5142 (PMC466958; doi:10.1126/sciadv.adn5142)
Supplement: Supplementary file 1 — Text S1 to S7 Figs. S1 to S15 Tables S1 and S2 [file sciadv.adn5142_sm.pdf]

Science Advances
AAAS

Supplementary Materials for

Highly stretchable dynamic hydrogels for soft multilayer electronics

Stephen J. K. O'Neill *et al.*

Corresponding author: Oren A. Scherman, oas23@cam.ac.uk

Sci. Adv. **10**, eadn5142 (2024)
DOI: 10.1126/sciadv.adn5142

This PDF file includes:

Text S1 to S7
Figs. S1 to S15
Tables S1 and S2

S.1 Preparation of Supramolecular Polymer Networks

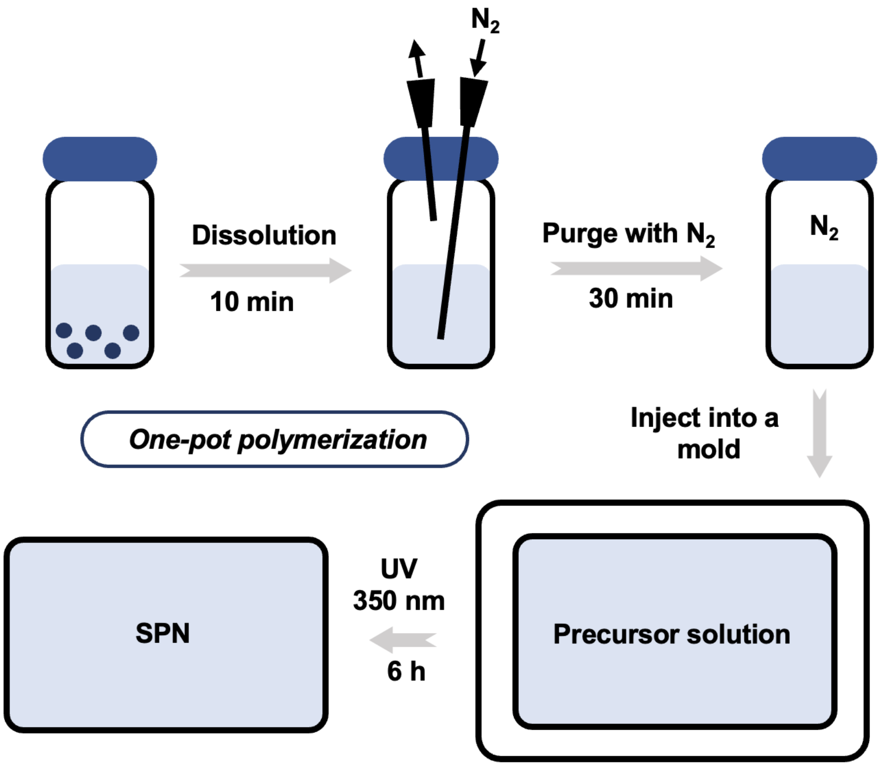

Figure S1: Schematic representation of the fabrication protocol of the supramolecular poly(ionic) networks. The precursor solution is injected into a glass mold for free-radical polymerization.

Figure S1 shows the six steps for the preparation of SPNs;

(1) Certain amounts of ionic monomer, non-covalent crosslinker (2BPyVI-CB[8], and photoinitiator (I-2959) were pre-determined and dissolved in Milli-Q water under ultrasonication for 10 min.
(2) The obtained precursor solution was sealed and purged with nitrogen for at least 30 min to remove oxygen in the solution phase that may eliminate radicals during polymerization.
(3) The precursor solution was carefully injected into a laboratory-made, non-stick glass mold until the whole mold was filled without any bubbles or spare space inside.
(4) The glass mold filled with the precursor solution was exposed to UV irradiation at 350 nm with 4.8 mW/cm^2 for 6 h to undergo *in situ* photo-polymerization in one pot.
(5) After *in situ* polymerization, the SPNs was removed from the glass mould and further cut into the test specimens with different sizes and shapes using a dumbbell/cylinder-shaped cutter or a

razor blade.

S.2 Design & Characterization of Guest Monomer BPyVI

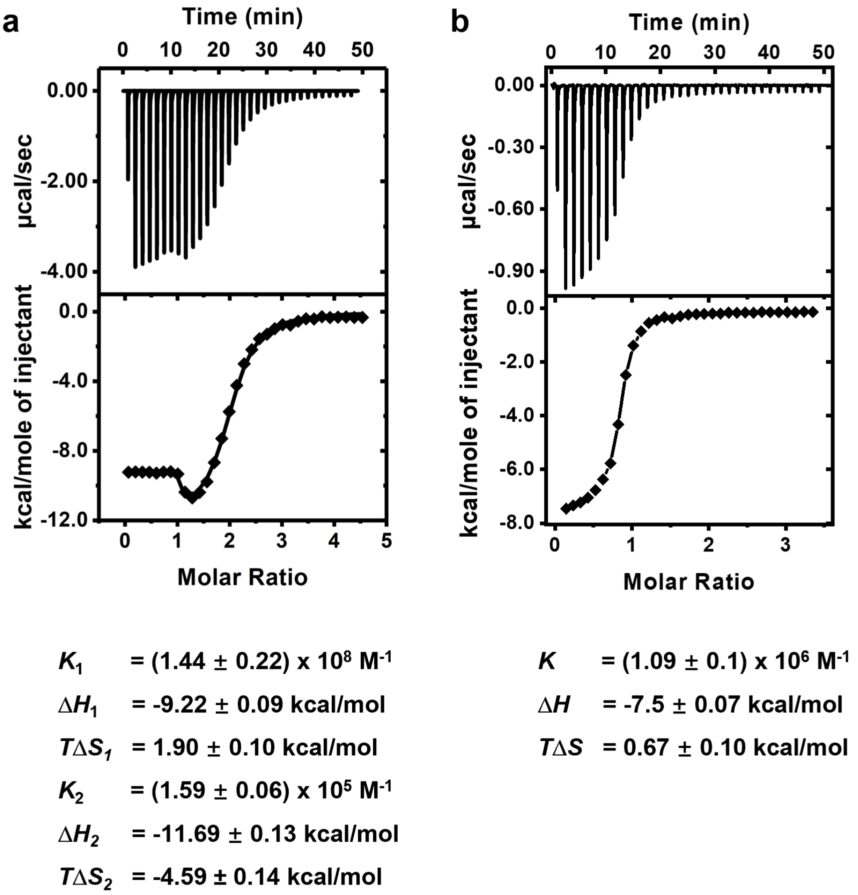

Figure S2: Thermodynamic investigation of BPyVI guest with CB[8] and CB[7] hosts. ITC titration plots and fitted curves obtained by titrating BPyVI guest with a) CB[8], and b) CB[7] at 298.15 K. A typical 2:1 binding ratio is observed in the case of CB[8], while 1:1 is observed for CB[7].

Table S1 below shows a variety of concentrations of the ternary complex for both BVI and BPyVI. While the solubility of CB[8] in the 2BVI-CB[8] complex is limited to below 50 mM, the 2BPyVI-CB[8] ternary complex can dissolve a CB[8] concentration of up to 150 mM, on account of the additional positive charges present on the BPyVI in the complex.

Table S1: Summary of the solubility of the 2BPyVI-CB[8] ternary complex. Table comparing solubility of 2BVI-CB[8] ternary complex, 2BPyVI-CB[8] ternary complex, a dimethyl adamantine (DMADA)-CB[8] complex, and the CB[8] alone. The photos of the corresponding solutions are shown above. Counterions have been omitted for clarity.

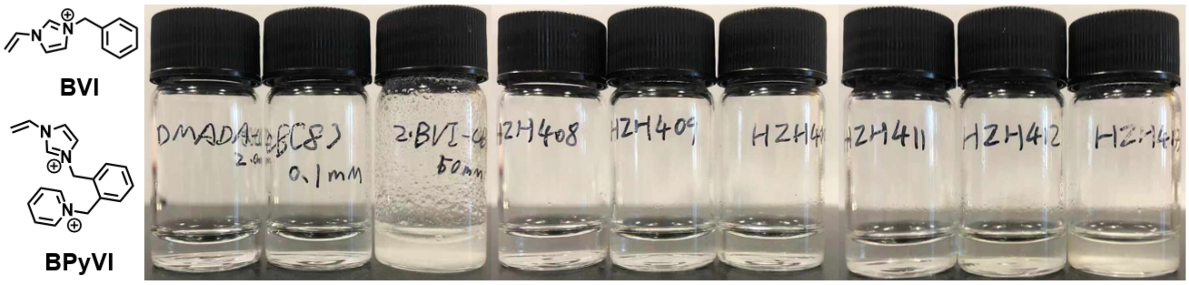

Conc.	1	2	3	4	5	6	7	8	9
CB[8]	2 mM	0.1 mM	50 mM	25 mM	50 mM	75 mM	100 mM	125 mM	150 mM
	2.6 mg	0.13 mg	65 mg	32.5 mg	65 mg	98 mg	130 mg	163 mg	195 mg
Guest	DMADA	---	BVI	BPyVI					
	2 mM	---	100 mM	50 mM	100 mM	150 mM	200 mM	250 mM	300 mM

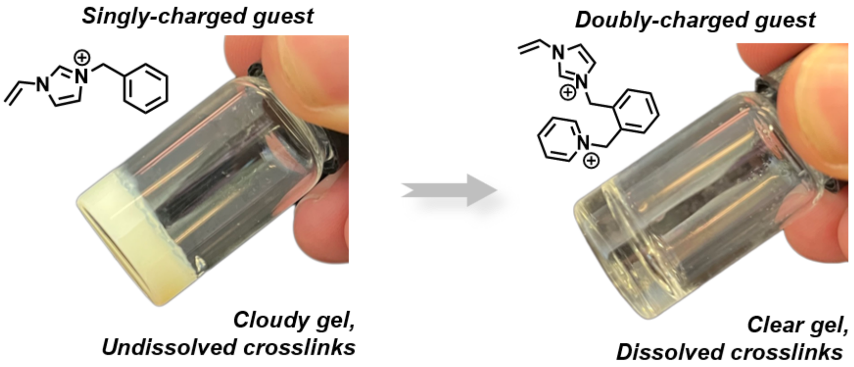

Figure S3: Guest molecule comparison. Copolymerization of SPAPS ionic monomer with singly (BVI) and doubly (BPyVI) cationic charged guest molecules. The additional positive charged on the guest molecules allows for full dissolution and integration of crosslinks into the network. Counterions are omitted for clarity.

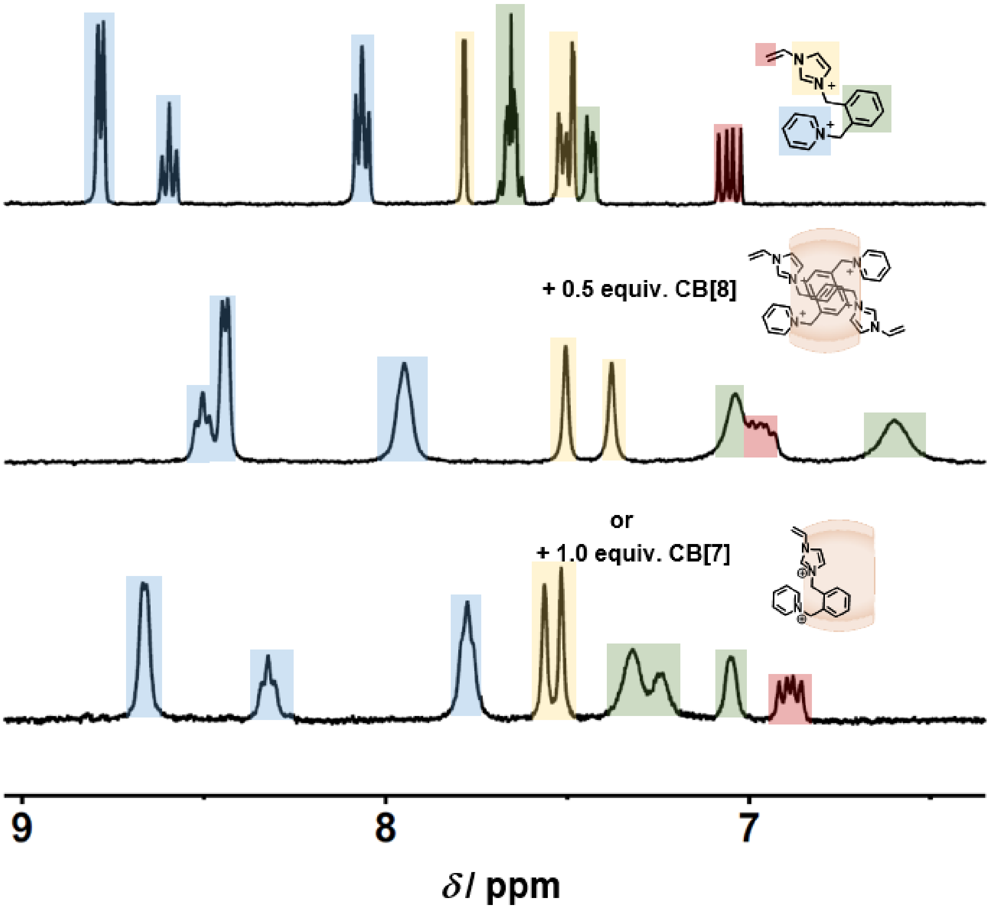

Figure S4: Characterization of BPyVI, 2BPyVI-CB[8] ternary complex and BPyVI-CB[7] complex. ^{1}H NMR spectra (D_2O, 298 K) of BPyVI (2 mM, top), 2BPyVI-CB[8] (1 mM, middle) and BPyVI-CB[7] (1 mM, bottom). Counterions have been omitted for clarity.

The ^{1}H NMR spectra of BPyVI, 2BPyVI-CB[8] and BPyVI-CB[7] are shown in Figure S4, from which it can be seen that the ^{1}H NMR spectra of the BPyVI guest alone and in the presence of CB[7] and CB[8] differ. Upon binding, the part of the guest molecule that binds within the host molecule cavity will experience more shielding, resulting in an upfield shift of such proton peaks. It can be seen that peaks corresponding to the phenyl protons, as well as the methylene protons, all exhibit an upfield shifts in the case of both CB[8] and CB[7].

S.3 Viscoelastic & Electromechanical Property Testing

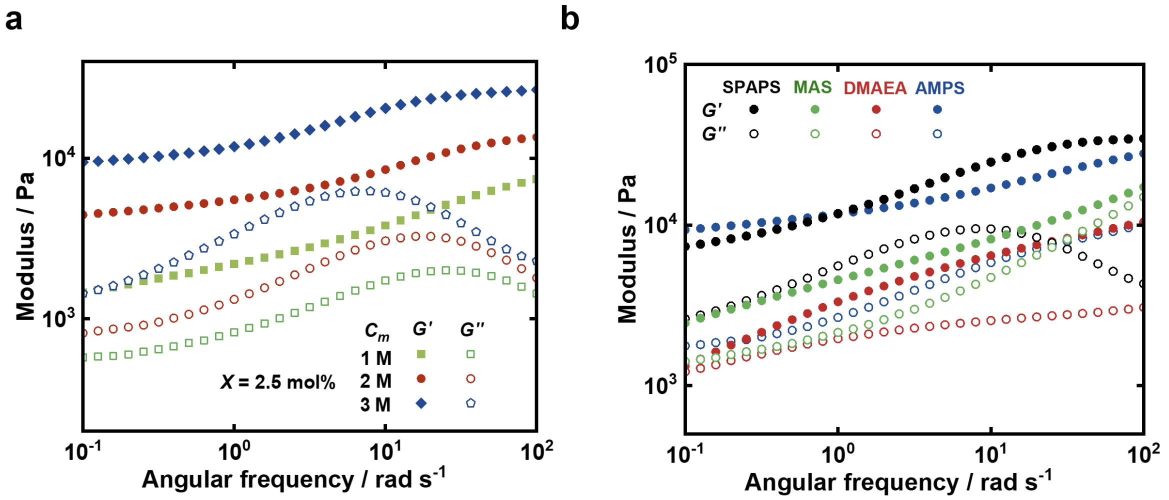

Figure S5: Viscoelastic characterization of supramolecular poly(ionic) networks. Plots of storage *G'* and loss *G"* modulus obtained by frequency-sweep measurements at a strain of 1%. a) Varying the concentration of SPAPS monomer, keeping the crosslink molar ratio (X) constant at 2.5%. b) Plot showing the full frequency sweep for each of the different ionic monomers.

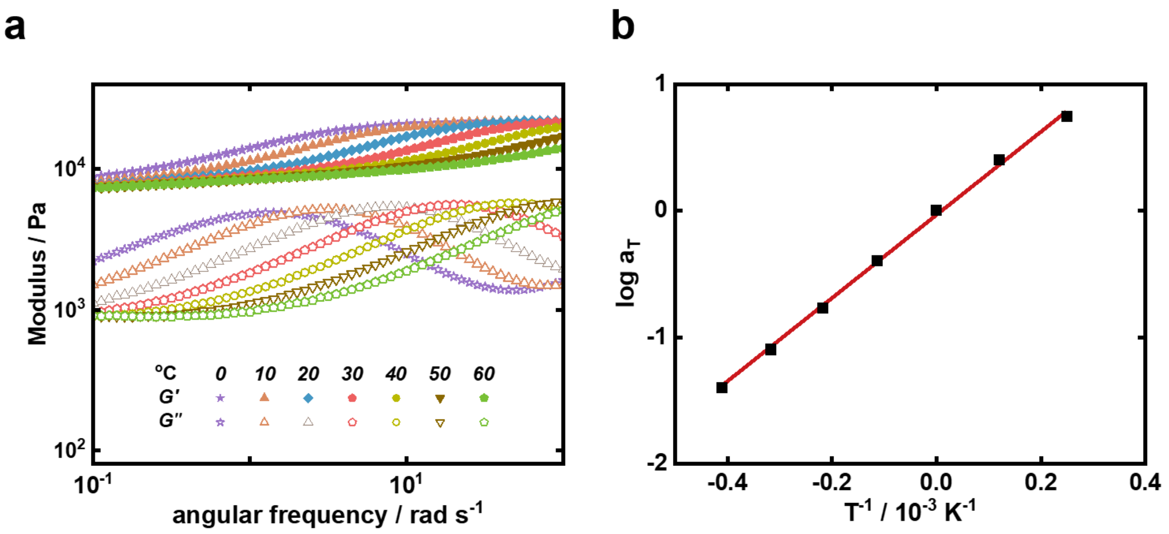

Figure S6: Time-temperature superposition experiment (TTS) for the supramolecular poly(ionic) networks. a) TTS data in which frequency sweep measurements were conducted at a variety of temperatures. b) Plot of the linear shift parameter a_T against the inverse temperature (K^{-1}).

Time-temperature superposition principle

The shift factors can be correlated with the temperature using the Arrhenius equation as follows:

$$log(a_{Tref}) = \frac{E_a}{2.303R}\left(\frac{1}{T} - \frac{1}{T_{ref}}\right)$$

where E_a is the activation energy in kJ mol^{-1} and R is the gas constant (8.314 J K^{-1} mol^{-1}). The shift factors can be fitted linearly to the inverse of temperature (1/T). The activation energy is a measure of the energy barrier that must be overcome to allow for the relaxation of the viscoelastic SPIN.

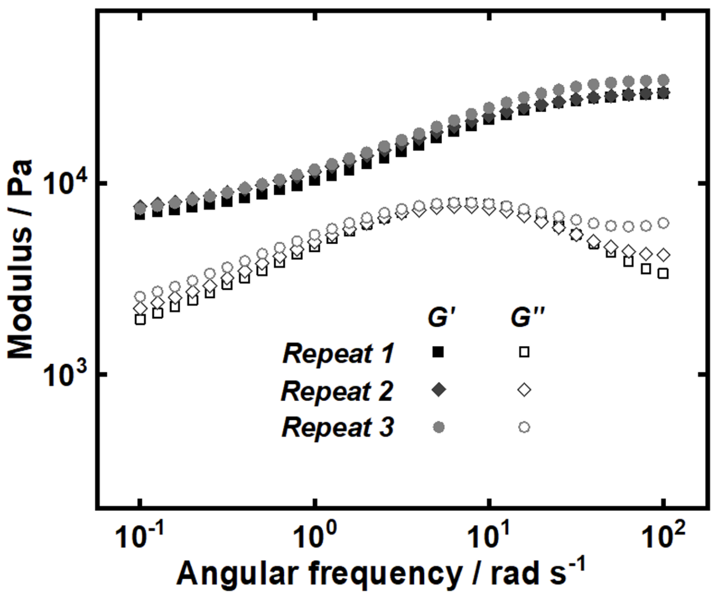

Figure S7: Viscoelastic characterization of supramolecular poly(ionic) networks. Plots of storage *G'* and loss *G"* modulus obtained by frequency-sweep measurements at a strain of 1% with a SPAPS network at C_m = 3.0 M with 2.5 mol% 2BPyVI-CB[8] repeated with three different samples.

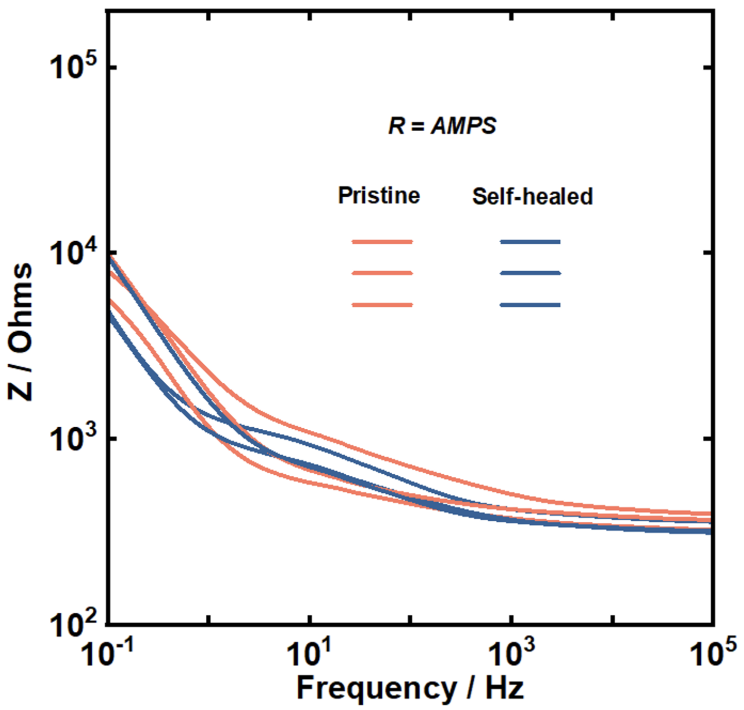

Figure S8: Electrochemical impedence spectroscopy of supramolecular poly(ionic) networks. Typical Impedance plots of an AMPS network at C_m = 3.0 M with 2.5 mol% 2BPyVI-CB[8] crosslinks repeated with three different samples.

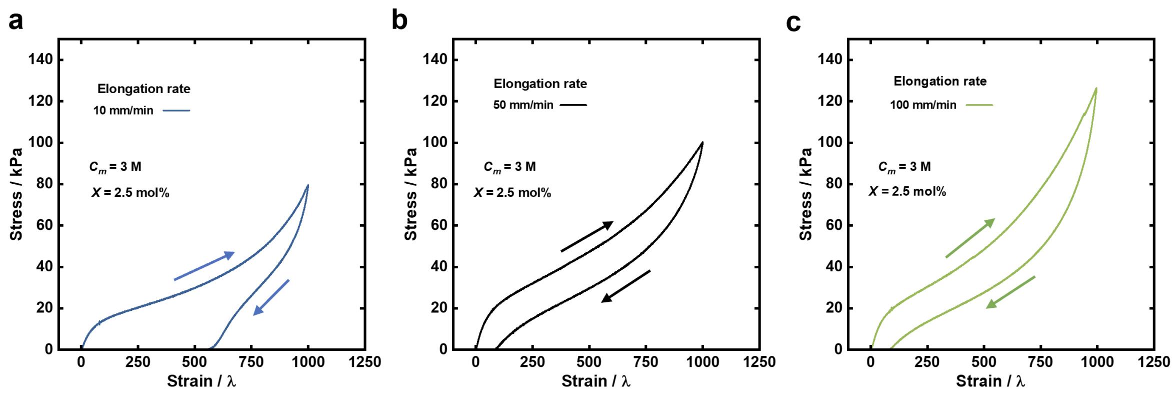

Figure S9: Hysteresis testing under tensile deformation at varying rates. Stress-strain curves at varying rates of deformation for a SPAPS network at C_m = 3.0 M with 2.5 mol% 2BPyVI-CB[8] to 1000% strain. a) Under a strain rate of 10 mm/min. b) Under a strain rate of 50 mm/min. c) Under a strain rate of 100 mm/min.

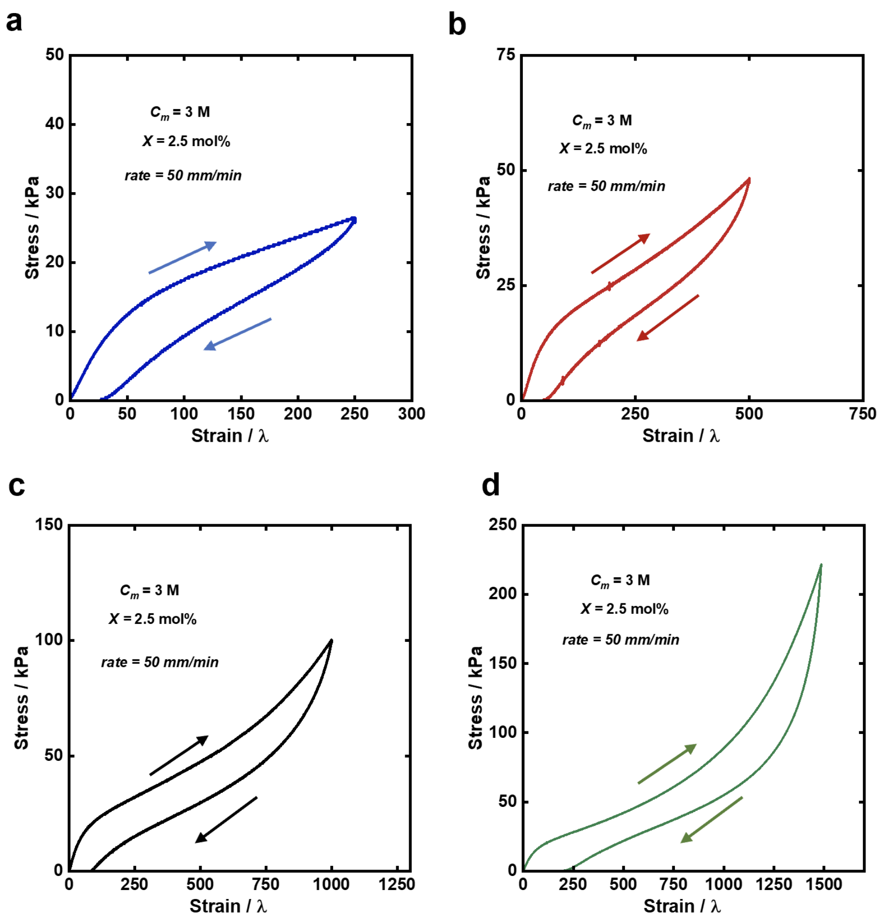

Figure S10: Hysteresis testing under tensile deformation to varying strains. Stress-strain curves to different degrees of deformation for a SPAPS network at C_m = 3.0 M with 2.5 mol% 2BPyVI-CB[8]. a) To 250% strain. b) To 500% strain. c) To 1000% strain. d) To 1500% strain.

As shown in Figure S9, at the slower rate of extension and retraction of 10 mm/min, there is a greater degree of hysteresis compared to the 50 mm/min and 100 mm/min. This is due to the viscoelastic nature of the supramolecular networks. When under applied strain, the polymers self-relax on account of the dissociation and re-association of the dynamic crosslinks. The longer that the polymer network is held at strain, the greater the degree of polymer rearrangement. Similarly as shown in Figure S10, there is an increase in the degree of hysteresis at larger deformations due to a greater degree of crosslink dissociation and reassociation while under extension, giving more polymer rearrangement.

S.4 High and Low Salinity Hydrogels

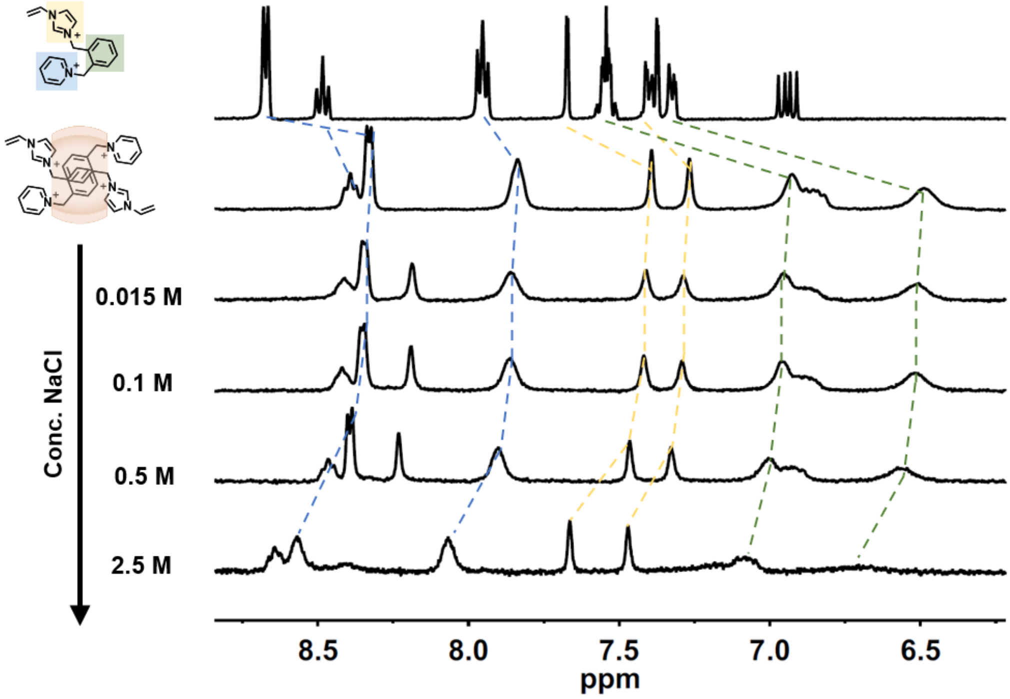

Figure S11: ^{1}H NMR characterization of the host-guest complex with NaCl. ^{1}H NMR of the 2BPyVI-CB[8] host-guest ternary complex with increasing NaCl concentration from 0.015 to 2.5 M. As the salt concentration is increased, the peaks corresponding to the guest shift back downfield and become broader, suggesting competitive binding between the guest and ionic species. Counterions have been omitted for clarity.

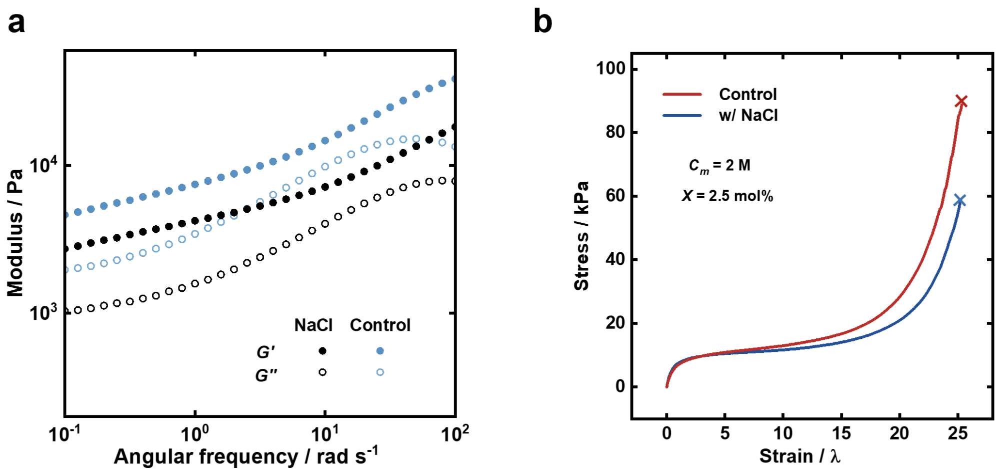

Figure S12: Characterization of high and low salinity supramolecular acrylamide hydrogels. a) Plots of storage *G'* and and loss *G"* modulus obtained by frequency-sweep measurements. b) Plots of the tensile test measurements. The high salinity hydrogel contains 2.5 M NaCl, while the low salinity hydrogel contains 0.015 M NaCl.

Figure S12 shows the viscoelastic and mechanical properties of the supramolecular polymer networks with and without NaCl. A lower modulus can be observed for the sample containing NaCl, which can be explained by the effect of NaCl on the binding dynamics of the 2BPyVI-CB[8] crosslinks, as shown in Figure S11. However, the NaCl containing networks remain highly stretchable (∼25x extensibility) with high tensile strength (∼60 kPa).

S.5 Anionic Selective Gel Mechanical Properties

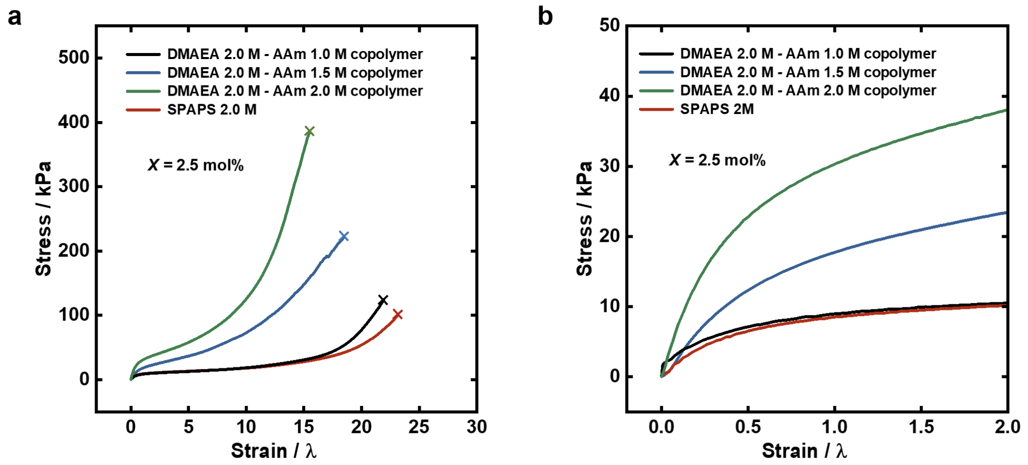

Figure S13: The affects of AAm copolymerization on DMAEA supramolecular network mechanical properties. a) Full stress-strain curves of DMAEA-AAm copolymers with varying concentrations of AAm monomer, compared to the 2 M SPAPS homopolymer network. b) Stress-strain curves across physiologically relevant strains.

Figure S13 shows the effect of copolymerization of DMAEA (2 M) with various concentrations of AAm (1–2 M). Upon the addition of 1 M AAm, the tensile properties become comparable to the homopolymer SPAPS at 2 M, especially at physiological levels of strain (*i.e.* $\lambda < 0.5$). This is an important factor in order to ensure that strain is applied evenly across the hydrogel power source with the addition of stress.

S.6 Tensile Property Testing

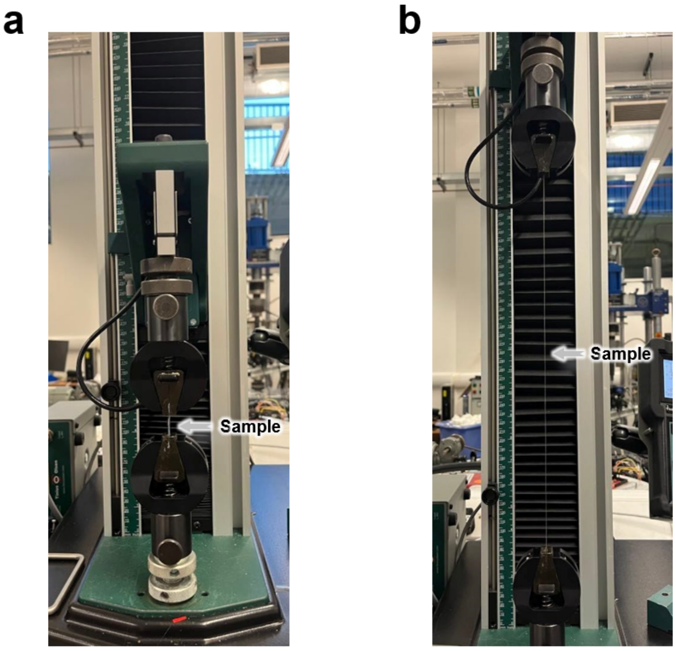

Figure S14: Experimental setup for tensile testing. Photographs of the experimental setup for tensile testing. The samples were cut into a dumbbell-shaped specimen (following ISO4661-1 standard), fixed at each end using self-tightening clamp grips, and stretched at a constant rate of 10 mm min^{-1} unless otherwise stated, to obtain the stress-strain curve. a) Photograph at the start of the tensile test, showing the unstretched sample. b) Photograph during the tensile test, showing the sample under strain.

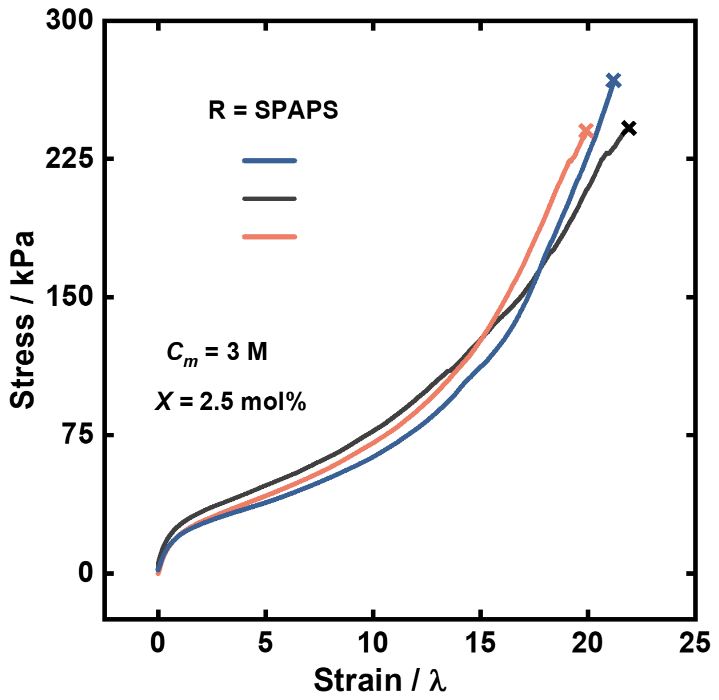

Figure S15: Tensile mechanical characterization of supramolecular poly(ionic) networks. Typical stress-strain curves obtained from tensile tests on a SPAPS network at C_m = 3.0 M with 2.5 mol% 2BPyVI-CB[8] crosslinks repeated with three different samples.

S.7 Comparison to Reports of Ionically Conductive Hydrogels

Table S2: Table Comparison to Reports of Ionically Conductive Hydrogels. Table comparing ionic conductivity, stretchability and Young's modulus of the SPINs compared to previous reports of ionically conductive polymeric hydrogels.

**Polymer**	**Ionic Conductivity** $\mathbf{S\ m^{-1}}$	**Stretchability** λ	**Reference**
PVA/NaCl (5 M)	3.4	5.2	38
PVA/NaCl (3 M)	2.5	8.0	38
PS/PEO	1	3.5	39
PEG/PAMAA-Fe^{3+}	0.6	13.5	40
PEG/PAAm/PAA	1	11	41
PAA/AETA	0.9	3.4	42
PAAm/CNF/KCl	0.5	4.3	43
PAAm/Gelatin	1.5	8.5	44
PAAm/PAA/FeCl	0.31	4.5	45
PEO/PAA	0.04	1.53	46
P(AAm-BA-ACG)	1.35	6.9	47
PAA/KCl/IL	0.8	7.1	48
**SPAPS**	**4.0**	**21.2**	**this work**
**AMPS**	**12.9**	**18.8**	**this work**
**DMAEA**	**2.9**	**16.3**	**this work**
**MAS**	**0.35**	**16.0**	**this work**

PVA = Polyvinyl alcohol, PS = Polystyrene, PAAm = Polyacrylamide, PAA = Polyacrylic acid, CNF = Cellulose nanofibers, PEO = Polyethylene oxide, PEG = Polyethylene glycol
